# Redox Activity of Copper(II) Complexes with NSFRY Pentapeptide and Its Analogues

**DOI:** 10.1371/journal.pone.0160256

**Published:** 2016-08-12

**Authors:** Magdalena Zofia Wiloch, Urszula Elżbieta Wawrzyniak, Iwona Ufnalska, Grzegorz Piotrowski, Arkadiusz Bonna, Wojciech Wróblewski

**Affiliations:** 1 Department of Microbioanalytics, Faculty of Chemistry, Warsaw University of Technology, Noakowskiego 3, 00–664, Warsaw, Poland; 2 Faculty of Chemistry, University of Gdańsk, Wita Stwosza 63, 80–308, Gdańsk, Poland; 3 Department of Biophysics, Institute of Biochemistry and Biophysics, Polish Academy of Sciences, Pawińskiego, 5a, 02–106, Warsaw, Poland; Indiana University School of Medicine, UNITED STATES

## Abstract

The influence of cation-π interactions on the electrochemical properties of copper(II) complexes with synthesized pentapeptide C-terminal fragment of Atrial Natriuretic Factor (ANF) hormone was studied in this work. Molecular modeling performed for Cu(II)-NSFRY-NH_2_ complex indicated that the cation-π interactions between Tyr and Cu(II), and also between Phe-Arg led to specific conformation defined as peptide box, in which the metal cation is isolated from the solvent by peptide ligand. Voltammetry experiments enabled to compare the redox properties and stability of copper(II) complexes with NSFRY-NH_2_ and its analogues (namely: NSFRA-NH_2_, NSFRF-NH_2,_ NSAAY-NH_2_, NSAAA-NH_2_, AAAAA-NH_2_) as well as to evaluate the contribution of individual amino acid residues to these properties. The obtained results led to the conclusion, that cation-π interactions play a crucial role in the effective stabilization of copper(II) complexes with the fragments of ANF peptide hormone and therefore could control the redox processes in other metalloproteins.

## 1. Introduction

Inorganic ions are necessary in living organism for vital cellular activity. They take part in a lot of processes for example in photosynthesis, cellular respiration and in the transmission of nerve impulses. The metal ions remain rarely in the free form, since they are complexed by proteins, peptides or amino acids. Many metalloproteins are metal-dependent enzymes, which proper and biologically active structure is reached through specific interactions with selected metal ions.

The metal ion binding occurs via a coordination bond, where the protein provides a metal chelating ligand. Statistics shows that copper and iron ions are generally coordinated by nitrogen atoms but also by sulfur and/or oxygen atoms belonging to the amino acid residue of the protein; magnesium and manganese ions are coordinated by oxygen, whereas zinc ions by nitrogen, oxygen and sulfur centers equally [1]. Another interesting feature of the metal-binding site is its surrounding i.e. the close environment of the metal ion is hydrophilic with a further hydrophobic sphere, composed of the aromatic rings of tryptophan (Trp), tyrosine (Tyr) and phenylalanine (Phe) residues [2]. Some reports suggest that the aromatic residues and the observed cation-π interactions could be directly responsible for the control of the redox properties of such complexes [3,4].

A cation-π involves interaction of π electrons from multiple bonds (e.g. aromatic rings) with various inorganic cations such as Cu^2+^, Ca^2+^, K^+^ [5,6] as well as organic cations such as guanidine group of arginine (Arg) and amino group of lysine (Lys) [7]. Cation—π interactions were discovered not only in chemistry, but also in biology [5,6] and It is commonly known that they play an important role in the stabilization of protein structures and alter the ligand-receptor affinity [8–11]. However, our knowledge on the mechanism of redox process control through cation-π interactions is limited. Studies concerning vitamin B_13_ demonstrated the ability to control the oxidation state of the copper ions through cation-π interactions [12]; nevertheless, the most interesting reports in this field describe the properties of the Cu(II)-NSFRY complexes [13–15].

The NSFRY peptide (where N is Asparagine, S is Serine, F is Phenylalanine, R is Arginine and Y is Tyrosine) is the C-terminal fragment of the Atrial Natriuretic Factor (ANF), a peptide hormone produced in the heart atrial muscle cells and involved in the regulation of blood pressure. Physiological influence of low copper diet and its relationship with ANF peptide has been reported by Bhathena et al [16]. It was postulated that increased plasma ANF levels in male rats may indicate the severity of the cardiopathy observed in Cu(II) deficiency.

The structure of Cu(II)-NSFRY complex is similar to the structure of the presented above metalloprotein i.e. copper(II) ion is coordinated by four nitrogen atoms of the peptide backbone. Thus, the first domain that binds metal is hydrophilic and a second hydrophobic sphere around the coordination center is formed by two aromatic rings of phenylalanine and tyrosine residues. Circular dichroism spectra and potentiometric results, supported by NMR and calorimetric measurements, confirmed that N-terminal amino nitrogen from asparagine (Asn) is an anchoring point in coordination of Cu(II) ions. The alanine scanning revealed that 4N complexes could be stabilized by hydrogen bonding between polar atoms of 1 (Asn) and 5 (Tyr) residues [13]. The protonation constants of NSFRY analogues as well as the stability constants of their copper(II) complexes were determined using pH-metric titrations and compared with the stability of simple pentaalanine complex [11,12]. According to Janicka et al. the exceptional stability of such complexes resulted from the highly organized side-chain structure and cation-π interactions between the aromatic ring of tyrosine and copper ion [13]. However, the additional π interactions, that may be observed between phenylalanine and arginine enhancing the stability of Cu(II)-NSFRY complex, were not considered in this report. Finally, the possible occurrence of the interactions between the d electrons of the metal ion and the π ring system was also considered for Ni(II) complexes with NSFRY-NH_2_ [13].

In the present work, electrochemical studies on redox activity of copper(II) complexes with selected pentapeptide ligands: NSFRY-NH_2_, NSFRA-NH_2_, NSFRF-NH_2_, NSAAY-NH_2_, NSAAA-NH_2_ and AAAAA-NH_2_ formed under various pH, were reported. Particular emphasis was given to elucidate the impact of cation-π interactions on the complex structure and stability, since bulky side chains situated below or above coordinate plane may interact with metal ion. Molecular modeling was used to explain the arrangement of amino acids in the structure of 4N complexes.

## 2. Materials and Methods

### 2.1 Reagents and Materials

Inorganic salts: potassium nitrate, nitric acid, potassium hydroxide, copper(II) nitrate hydrate, hexaammineruthenium(III) chloride of the highest purity were supplied by Sigma-Aldrich and were used without further purification. All solutions used in electrochemical measurements were prepared with deionized water passed through a Milli-Q purification system (Millipore). The final resistivity of water was 18 MΩ cm^-1^. The pH of the prepared solutions was adjusted with submicroliter volumes of either KOH or HNO_3_ solutions using SevenCompact pH-meter (Mettler-Toledo) with InLab Micro Pro micro combination pH electrode (Mettler-Toledo). Glassware utilized in the experiments was rinsed with 6 M HNO_3_ followed by Milli-Q water before use to avoid Cu(II) contamination.

### 2.2 Molecular Modeling

Semi-empirical calculations were carried out using MOPAC2009TM. The geometry of Cu(II)-NSFRY-NH2 complex were fully optimized using PM6 Hamiltonian in Unrestricted Hartree-Fock approximation. In all calculations the electronic state of copper(II)-peptide complexes was assumed as a doublet. Ab initio (DFT) calculations were performed using the Gamess(US) package. The geometries were optimized using B3LYP as implemented in GAMESS(US) with 6-31G(d) basis sets.

### 2.3 Peptide Design

NSFRY-NH_2_ peptide was chosen as an approximate model of metal ion-binding site in proteins. The first modification was carried out in the fifth position (substitution of tyrosine by alanine); the NSFRA-NH_2_ peptide was designed to study the influence of Tyr residue and cation-π interactions on the redox properties of the species. Next, the NSFRF-NH_2_ derivative (substitution of tyrosine by phenylalanine) was designed to evaluate the effect of the removal of hydroxyl group on Cu(II)-aromatic ring cation-π interactions. Another change was the substitution of the third and fourth residues (phenylalanine and arginine) by alanine–peptide NSAAY-NH_2_ synthesized in order to investigate the impact of intramolecular cation-π interaction between Phe and Arg on the redox activity of the origin compound. In addition, the properties of Cu(II) complex of AAAAA-NH_2_ and NSAAA-NH_2_ were studied and compared with our peptide model.

### 2.4 Peptide Synthesis

Peptides NSFRY-NH_2_, NSFRA-NH_2_, NSFRF-NH_2_, NSAAY-NH_2_, NSAAA-NH_2_ and AAAAA-NH_2_ were synthesized automatically on the Prelude Peptide Synthesizer (Protein Technologies, Inc.) according to the Fmoc strategy [14]. Purification of the final products was accomplished by HPLC and pure lyophilized peptides were analyzed by ESI-Q-TOF Premier mass spectrometer to confirm their correct molecular masses.

### 2.5 UV–Vis Spectroscopy

UV-Vis spectra were recorded at 25°C on Cary 60 spectrophotometer (Agilent), over the spectral range 250–900 nm and with the optical path 1 cm. UV-Vis methods were used to control the real concentration of copper(II) and peptide solutions. The concentration of stock solutions of AAAAA-NH_2_ were determined by Cu(II) titrations, whereas the concentration of the solution of NSFRY and its analogues were evaluated on the basis of the molar extinction coefficient of tyrosine at 276 nm (ε = 1410 M^-1^ cm^-1^, pH = 7.4) [15] or phenylalanine at 257.5 nm (ε = 195 M^-1^ cm^-1^) [17].

### 2.6 Voltammetry

The electrochemical experiments were done in a three-electrode arrangement with a silver/silver chloride (Ag/AgCl) as the reference, platinum foil as the counter and glassy carbon electrode (GCE, BASi, 3 mm diameter) as the working electrode. The reference electrode was separated from the working solution by an electrolytic bridge filled with 0.1 M KNO_3_ solution. The potential of the reference electrode was calibrated by using ruthenium electrode process in the same electrolyte solution. The GC electrode was sequentially mechanically polished with 1.0, 0.3 and 0.05 μm alumina powder on a Buehler polishing cloth to a mirror-like surface. In order to remove remaining powder, the electrode has been sonicated for 1 min in water. After this procedure, the substrates were transferred to the sample solutions.

All electrochemical measurements were carried out in 96 mM KNO_3_ solution containing 4 mM HNO_3_; the concentration of peptides was 0.5 mM and the ligand-to-copper(II) ratio was 1:0.9 (small Cu(II) deficiency was applied to avoid the interference from uncomplexed Cu(II) cations). The pH was adjusted by adding a small volume of concentrated KOH and HNO_3_ solutions. The pH was closely controlled before, during and at the end of each voltammetric measurement. Cyclic (CV) and differential pulse voltammetry (DPV) were performed using the CHI 1030 potentiostat (CH Instrument, Austin, USA). For all presented CV curves, the scan rate (*v*) was 100 mV/s. The following parameters were used in DPV technique: amplitude (ΔE) 50 mV, pulse time (tp) 10 ms. The acquisition of voltammetric curves was repeated 9 times for each solution of Cu(II)-peptide complex. Argon was applied to deaerate the solution and argon blanket was maintained over the solution during the experiments carried out at 25°C.

## 3. Results and Discussion

### 3.1 Molecular Modeling and Peptide Design

The first step of our study was to calculate the structure of Cu(II)-NSFRY-NH_2_ complex with the minimum energy state. Janicka et al. performed this task but, in our opinion, the authors did not consider a very important aspect [13]. On the basis of our previous study [18] a following hypothesis was put forward: the Phe and Arg residues in the Cu(II)-NSFRY-NH_2_ are involved in intramolecular cation-π interactions. Since these interactions may have an influence on the complex stability and its redox properties, the recalculation of the structure of the studied complex was carried out.

**Fig 1:**
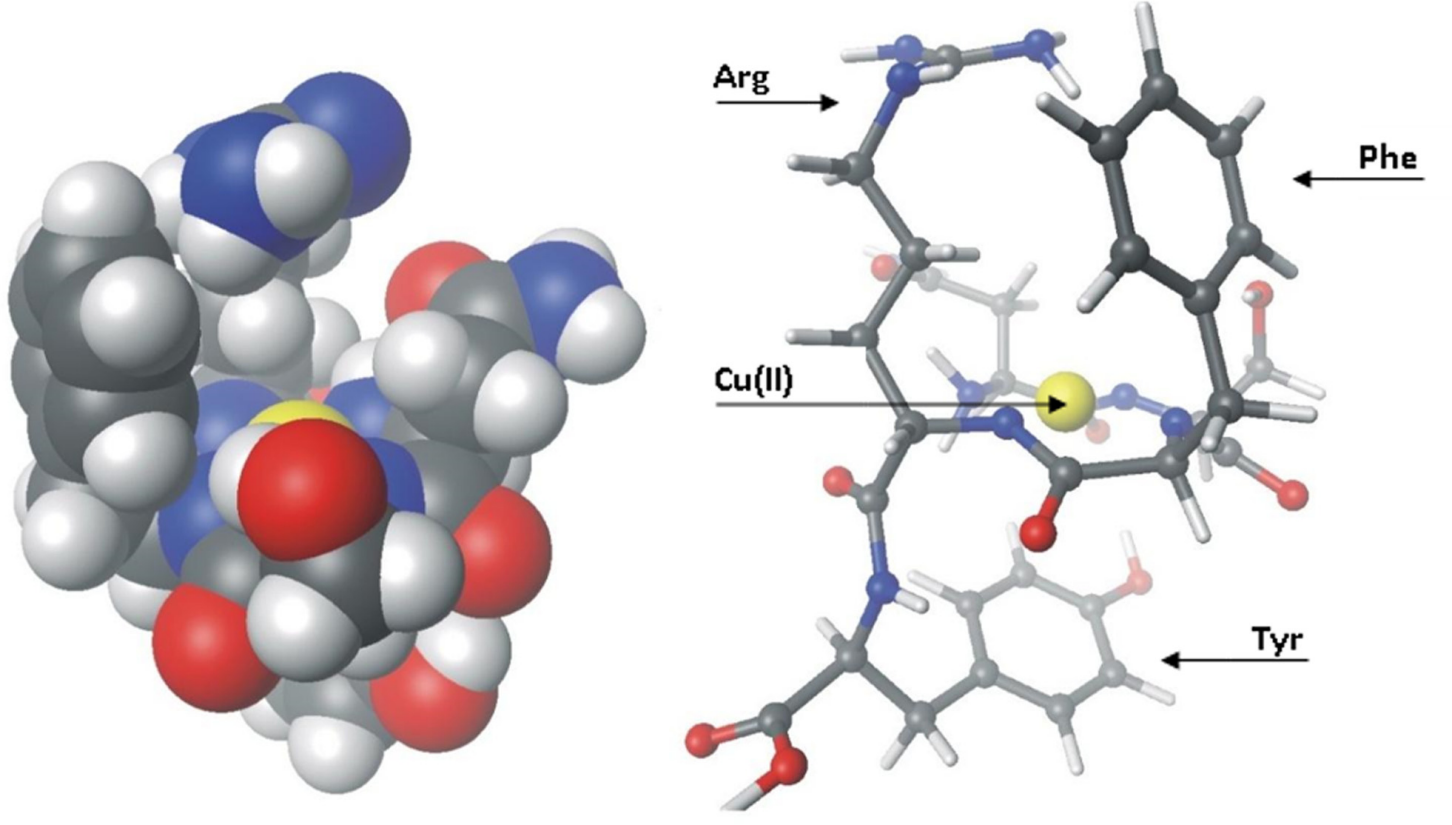
Calculated structures of the Cu(II)-NSFRY-NH_2_ complex. Ball model–left side–and the stick model–on the right side.

Structure, with the minimum energy, obtained from calculations (Fig 1) shows that, in fact, tyrosine residue from fifth position of peptide is below coordination sphere of the studied complex. Distance between the center of the aromatic ring of Tyr to the copper ion is 3.5 Å, which corresponds to the values reported in the literature [19,20], and it is the value characteristic for cation-π interaction. The close observation of the side chains of Phe and Arg clearly indicated that these two amino acids are above the coordination sphere. They are very close to each other and located in the vicinity of copper(II) ion. Moreover, the aromatic ring of Phe is closer to the guanidine group of the Arg than to the copper(II) ion. The minimum distance evaluated between these two groups is 3.25 Å. The ball model shows us that the aromatic ring of Phe is close enough to copper(II) ion to interact with it through the cation-π. In spite of all, the setting of the ring relative to the Cu(II) cation may impede such interaction. The aromatic ring of Phe, in contrast to the Tyr ring, is not parallel to the coordination plane which demonstrates that the Phe-Arg interaction is more probable. Aromatic ring of Tyr strongly reduces the contact of the copper(II) ion with the solvent. The same situation occurs above the coordination plane, with the one exception: in this case, the Phe-Arg pair creates a kind of protective, cation-π roof for copper(II) ion. Those structures (Fig 1), in which the metal cations are isolated from the solvent by peptide ligand were called peptide boxes [18]. It should be emphasized, that such specific structure may influence the redox properties of the metal-peptide complex.

According to the molecular modeling results, several pentapeptide ligands (namely: NSFRY-NH_2_, NSFRA-NH_2_, NSFRF-NH_2_, NSAAY-NH_2_, NSAAA-NH_2_ and AAAAA-NH_2_) were selected and synthesized to assess the impact of cation-π interaction on the stability and redox behavior of the metal-peptide complexes as well as to evaluate the contribution of individual amino acid residues on these properties.

### 3.2 Electrochemistry

The redox behavior of NSFRY-NH_2_ model ligand, its four analogues–NSFRA-NH_2_, NSFRF-NH_2_, NSAAY-NH_2_, NSAAA-NH_2_ and pentalanine AAAAA-NH_2_ as well as their complexes with copper(II) were investigated by cyclic voltammetry (CV) and differential pulse voltammetry (DPV). Electrochemical characterization of the most stable coordination structures (in short, 4N(O)^-^, 4N included in Table 1) and also other complex species, differing in the number of nitrogen atoms bound to the Cu(II) (Table 1–3N and 2N) was presented below. According to the species distribution diagrams [13], indicating the predominance of a given complex in various pH range, particular coordination forms were obtained by appropriate adjusting the pH of solution (see Table 1). Among the studied copper(II) complexes, particular emphasis was placed to study the redox properties of 4N structures exhibiting a very high thermodynamic stability [11].

**Table 1:** Various complex species formed at the appropriate pH (values given in brackets), determined on the basis of spectroscopic and potentiometric measurement [13] (the pH value for Cu(II)-NSAAY-NH_2_ 4N complex was estimated on the basis of UV-Vis spectra measured in the present work).

peptide	4N(O)^-^	4N	3N	2N	1N
**AAAAA-NH**_**2**_		CuH_-3_L (11.0)	CuH_-2_L (8.0)	CuH_-1_L (6.5)	CuL (5.5)
**NSFRY-NH**_**2**_	CuH_-3_L (11.0)	CuH_-2_L (9.0)	CuH_-1_L (6.7)	CuL (6.0)	CuHL (5.0)
**NSFRA-NH**_**2**_	-	CuH_-3_L (9.0)	CuH_-2_L (7.1)	CuH_-1_L (6.0)	CuL (5.0)
**NSAAY-NH**_**2**_	CuH_-3_L	CuH_-2_L (9.0)	CuH_-1_L	CuL	CuHL

#### 3.2.1. Voltammetry of 4N(O)^-^ complexes

**Fig 2:**
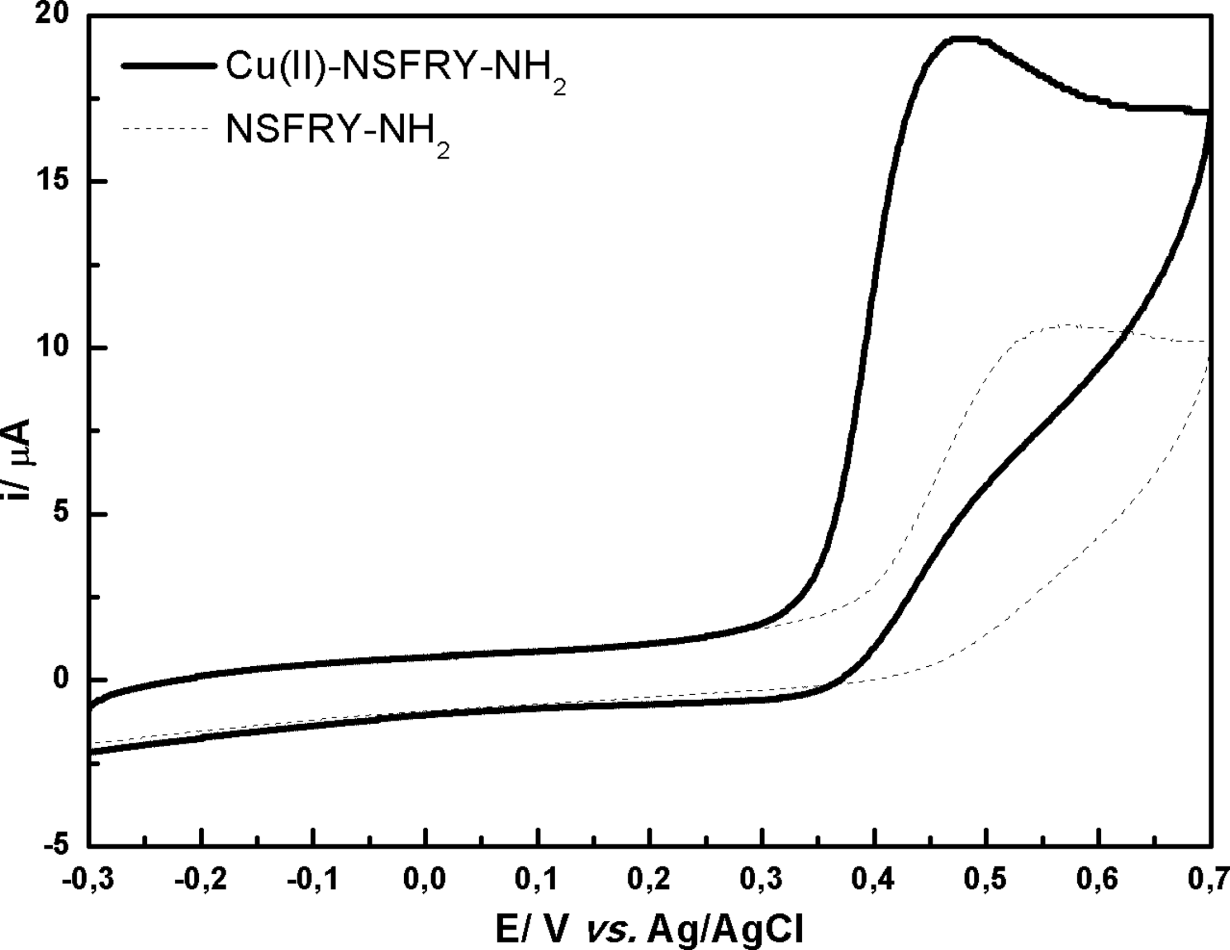
Cyclic voltammograms recorded in 0.5 mM aqueous solution of Cu(II)-NSFRY-NH_2_ complex (solid line) and NSFRY-NH_2_ peptide (dashed line) at pH 11.0.

The 4N(O)^-^ species exist in the case of Cu(II)-NSFRY-NH_2_ and Cu(II)-NSAAY-NH_2_ complexes, since the deprotonation of tyrosine is necessary for their formation. Fig 2 shows the comparison of cyclic voltammograms recorded in NSFRY-NH_2_ peptide solution and upon binding of Cu(II) ions. Tyrosine (Y) located in the fifth position of the N terminal peptide chain is a redox active amino acid and thus undergoes irreversible oxidation (Fig 2, dotted line). This process is pH-dependent and occurs more easily under alkaline conditions. The mechanism of tyrosine oxidation at carbon electrodes involves the formation of a thermodynamically unstable radical that stabilization leads to an electroactive orthoquinone structure [21].

Copper(II) coordinated by NSFRY-NH_2_ oligopeptide at pH = 11.0 (4N(O)^-^ forms according to the species distribution diagrams for Cu(II) complexes indicated by potentiometry [13]) is irreversibly oxidized to Cu(III) at 480 mV. Compared to the free NSFRY-NH_2_ ligand, the anodic peak appears at lower positive value (Fig 2). The lack of the cathodic peaks in the case of the complex suggests that the oxidized form of Cu(II)-NSFRY-NH_2_ is a catalyst for oxidation of tyrosine (i.e. Cu(III)-NSFRY-NH_2_ form simultaneously oxidizes tyrosine and restores the initial form). It is worth to note, that the catalytic oxidation of tyrosine in copper(II) complex was observed only at pH = 11.0, when tyrosine is deprotonated and 4N(O)^-^ species prevail.

#### 3.2.2. Redox properties of 4N complexes

The electrochemical behavior of Cu(II) bound to NSFRY-NH_2_ peptide is mainly influenced by the geometrical structure of the complex. Since all studied 4N copper(II) complexes have planar geometry, a Cu(II)/Cu(III) electrode process was predicted. The formal potential (E_f_) for such {NH_2_, 3N^-^} copper complex was estimated ~410 mV *vs*. Ag/AgCl [22–24]. Additionally, the presence of histidine (His) in the peptide sequence leads to a shift of E_f_ to more positive values: ~660 mV and ~770 mV *vs*. Ag/AgCl for {Im(His), 3N^-^} [25] and {NH_2_, Im(His), 2N^-^} [24] species, respectively. The oxidation of the studied Cu(II) complex proceeds according to the reaction:

$$\left[{\text{Cu}}^{\text{II}}{\text{H}}_{\text{-n}}\text{L}\right]\;\to \;{\left[{\text{Cu}}^{\text{III}}{\text{H}}_{\text{-n}}\text{L}\right]}^{+}+\text{e},$$

where n = 2 or 3 according to Table 1

The formal redox potentials and half-peak potentials for all studied electroactive species are collected in Table 2 (only the most stable structures were included).

**Table 2:** Potentials and peak width (calculated from CV curves), b_1/2_-peak width at half height from (calculated from DPV curves) for particular Cu(II)-peptide complexes. All values were given in [mV].

complex	E_a_	E_a/2_	E_a_-E_a/2_	E_c_	E_c/2_	E_c_-E_c/2_	ΔE	E_f_	b_1/2_
**Cu(II)-NSFRF-NH**_**2**_	538 ±1	479 ±2	59 ±2	472 ±3	415 ±3	-43 ±3	66 ±3	505 ±1	100 ±1
**Cu(II)-NSFRY-NH**_**2**_	518 ±2	460 ±1	58 ±2	450 ±2	498 ±3	-48 ±3	69 ±3	484 ±1	108 ±0
**Cu(II)-NSFRA-NH**_**2**_	507 ±2	444 ±5	63 ±4	438 ±2	489 ±2	-51 ±2	68 ±2	472 ±1	100 ±1
**Cu(II)-NSAAY-NH**_**2**_	503 ±3	440 ±3	63 ±3	417 ±4	470 ±3	-53 ±5	86 ±5	460 ±3	98 ±4
**Cu(II)-AAAAA-NH**_**2**_	457 ±9	375 ±4	81 ±6	307 ±16	375 ±14	-68 ±7	150 ±24	382 ±6	127 ±13

E_a_/ E_c_- anodic/cathodic peak potential

E_a/2_/ E_a/2_ –anodic/cathodic potential at which the current reaches half of the peak current

∆E—the difference between anodic and cathodic potential | E_a_—E_c_ |

Ef—formal potential

The most positive formal potential of the Cu(II)/Cu(III) redox couple is observed when copper cation is bound to NSFRY-NH_2_ ligand. Therefore, the Cu(II)-NSFRY-NH_2_ complex is relatively the most thermodynamically stable (more precisely, the ratio between the values of stability constants of Cu(II)-NSFRY-NH_2_ and Cu(III)-NSFRY-NH_2_ complexes determines the potential shift of the Cu(II)/Cu(III) couple; however, since the previous studies reported the highest stability of Cu(II)-NSFRY complexes the positive shift was attributed in our work to the stabilization of Cu(II)-NSFRY versus Cu(III)-NSFRY species). It may be caused by steric fitting of the coordination center by the amino acid side groups. Moreover, much attention should be paid to the additional cation-π interactions between copper(II) ions and amino acids having an aromatic ring i.e. tyrosine and phenylalanine. Two aromatic amino acids occurring in the peptide sequence have several possibilities to participate in this type of interactions: Tyr and Phe may interact with each other, with guanidine group of the side-chain of arginine, and also, sweep the area around the coordination center. Consequently, because AAAAA-NH_2_ ligand has neither aromatic nor large side substituents, the coordination center is associated only with 4 nitrogen atoms (one of the N-terminus and three from peptide bond). Therefore, the coordination center is not shielded, which facilitate the redox process at less positive potentials. On the other hand, the reversibility of studied redox processes should be considered carefully. The presented voltammograms show that the electron transfer is slow and redox process is the least reversible in the case of Cu(II)-AAAAA-NH_2_ (see values of ∆E and b_1/2_, Table 2). It suggests that a reorganization of this complex structure must occur (according to *square scheme of electrochemical* process) [26] to get its oxidized form. Indeed, copper complex with AAAAA-NH_2_ may be weak both in oxidized (Cu(III)-AAAAA-NH_2_) and reduced (Cu(II)-AAAAA-NH_2_) form.

**Fig 3:**
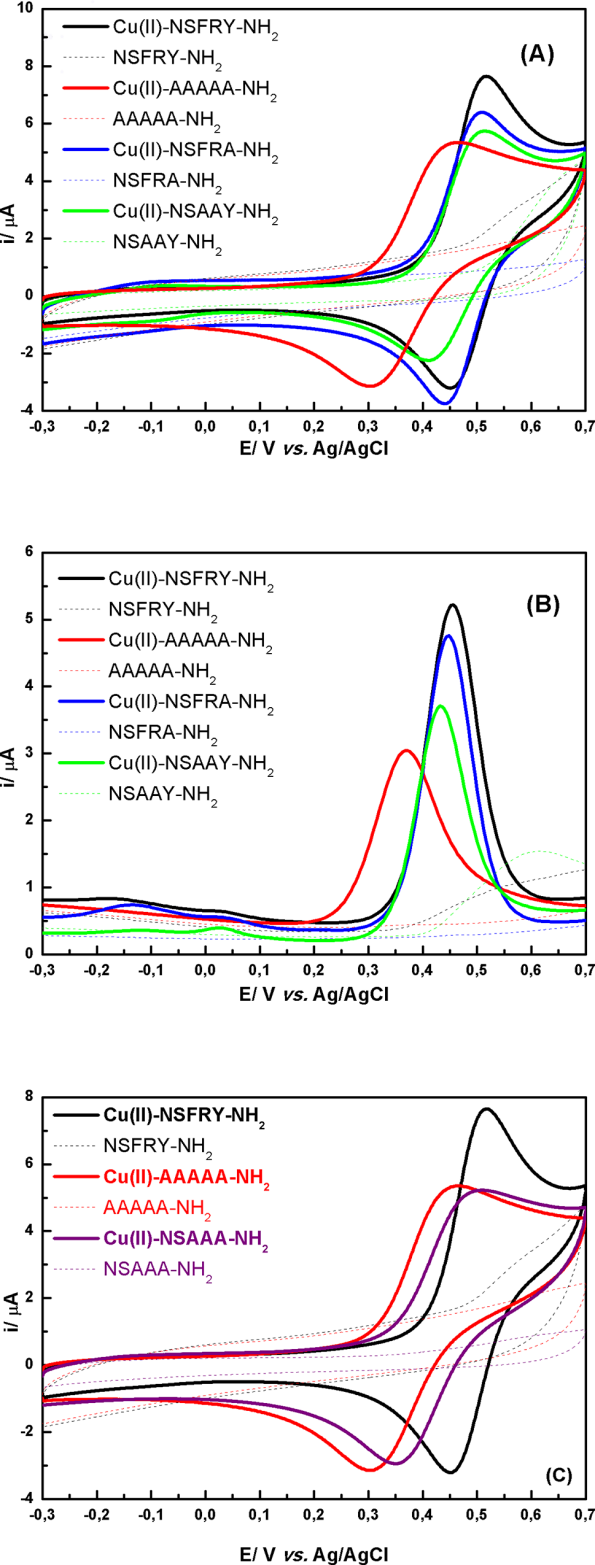
(A), (C) Cyclic and (B) DP voltammograms recorded in 0.5 mM aqueous solution of Cu(II)-peptide complexes (solid lines) and free peptides (dashed lines) at pH 9.0. Only in the case of Cu(II)-AAAAA-NH_2_ complex (red line) and AAAAA-NH_2_ peptide (red dashed line) the measurements were performed at pH 11.0.

Minor differences in position of oxidation peak were observed for: Cu(II)-NSFRA-NH_2_ and Cu(II)-NSAAY-NH_2_ complexes. The replacement of tyrosine in the fifth location of NSFRY-NH_2_ peptide chain by alanine eliminates the aromatic residue from the proximity of the coordination center and thus weakened cation-π interactions. Therefore, the peak potential of Cu(II)-NSFRA-NH_2_ is ca. 10 mV less positive than for Cu(II)-NSFRY-NH_2_ (Table 2, Fig 3A). Similarly, the introduction of alanine in the third and fourth position of peptide sequence (Cu(II)-NSAAY-NH_2_) increases the susceptibility of coordination center to oxidation, since the steric hindrance was reduced above the coordination plane. It is worth noting that the peaks current for reversible cyclic curves obtained when NSFRA-NH_2_ or NSAAY-NH_2_ coordinate the copper(II) ions decrease significantly with respect to the peak current recorded for Cu(II)-NSFRY-NH_2_ complex. DPV curves reflect more accurately the differences in redox properties of all studied complexes, resulting from their distinct stability, which is the consequence of inter- and intramolecular cation-π interactions (Fig 3B). However, it should be emphasized that the intensities of anodic peaks should be compared with caution since the contents of 4N form of particular Cu(II)-pentapeptide complexes are different in pH 9.0 (see potentiometric data [13]).

**Fig 4:**
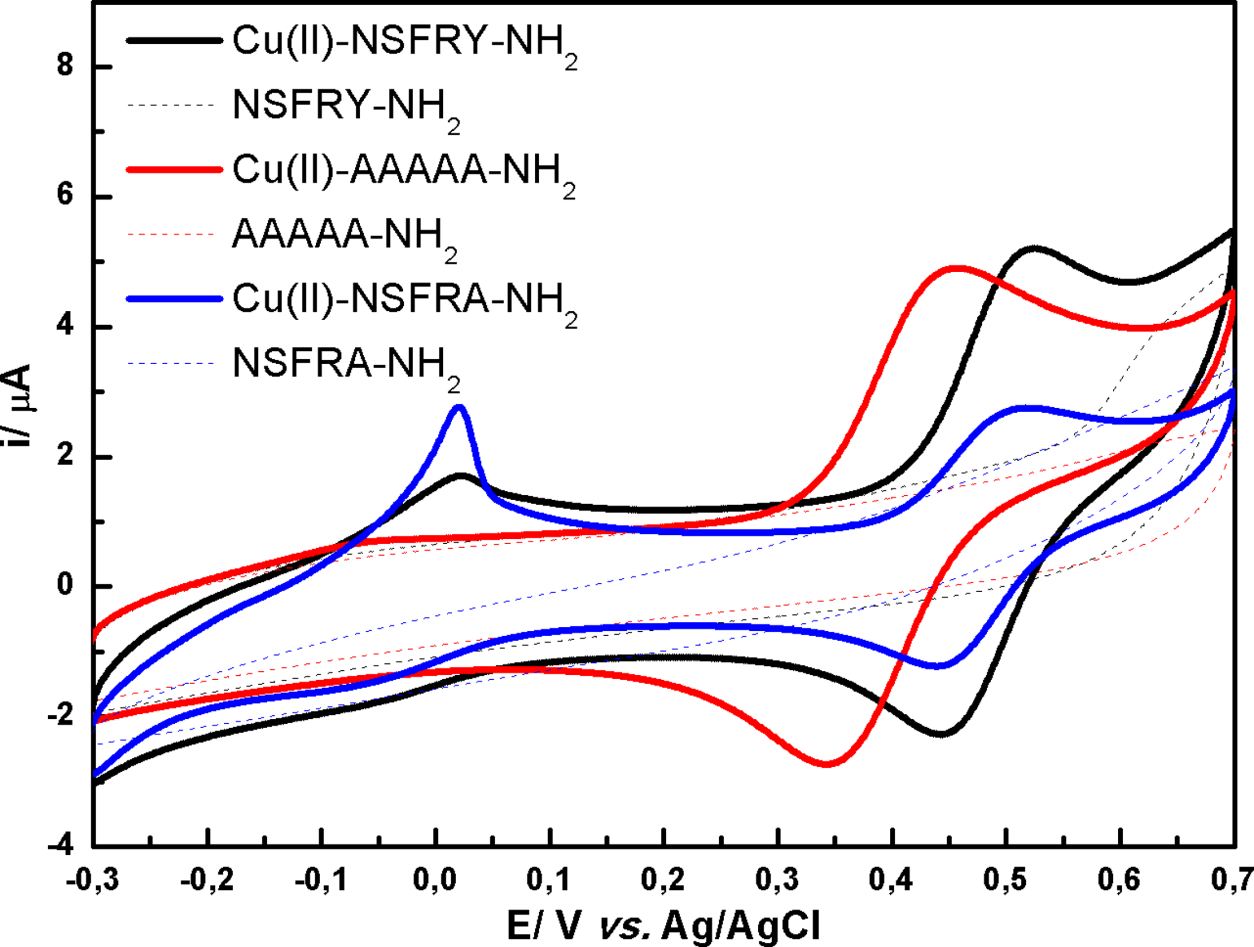
Cyclic voltammograms recorded in 0.5 mM aqueous solution of Cu(II)-NSFRY-NH_2_ complex (black solid line) and NSFRY-NH_2_ peptide (black dashed line) at pH 6.7; Cu(II)-AAAAA-NH_2_ complex (red solid line) and AAAAA-NH_2_ peptide (red dashed line) at pH 8.0; Cu(II)-NSFRA-NH_2_ complex (blue solid line) and NSFRA-NH_2_ peptide (blue dashed line) at pH 7.1.

**Fig 5:**
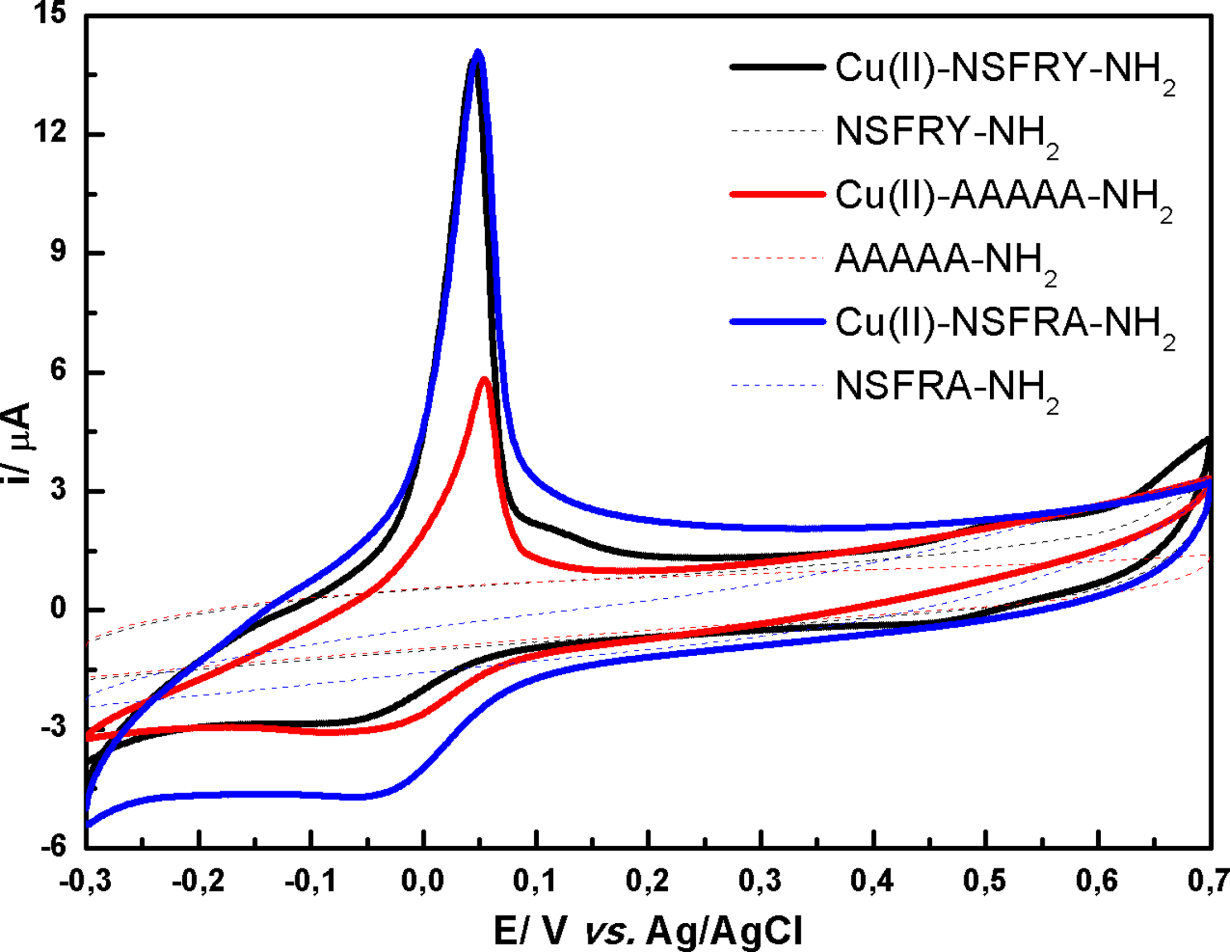
Cyclic voltammograms recorded in 0.5 mM aqueous solution of Cu(II)-NSFRY-NH_2_ complex (black solid line) and NSFRY-NH_2_ peptide (black dashed line) at pH 6.0; Cu(II)-AAAAA-NH_2_ complex (red solid line) and AAAAA-NH_2_ peptide (red dashed line) at pH 6.5; Cu(II)-NSFRA-NH_2_

Taking into account that NSAAA-NH_2_ derivative reflects the impact of the absence of cation-π interactions in relation to Cu(II)-NSFRY, CV curves recorded for Cu(II)-NSAAA-NH_2_, Cu(II)-AAAAA-NH_2_ and respective peptides were compared in Fig 3C. As could be expected, the formal potential estimated for Cu(II)-NSAAA-NH_2_ is more positive than for Cu(II)-AAAAA-NH_2_ and the reversibility of redox process is very similar due to the presence of Ala residues causing the reorganization of the complex structure. However, AAAAA-NH_2_ was chosen as a reference peptide (see Figs 3–5) according to many papers reporting the stability constants of metallocomplexes of NSFRY-NH_2_ and its analogues. Additionally, the effect of replacement of Tyr by Phe (NSFRF-NH_2_) on coordination and thermodynamic stabilization of the Cu(II)-NSFRF complex was studied. The deletion of the hydroxyl group from Tyr led to the increase of stability of the formed complex (see the value of formal potential in Table 2). We hypothesize, that the aromatic ring from Phe residue can be much closer to the copper(II) ion, providing optimum distance for cation-π interaction. Hydroxyl group of Tyr is engaged in hydrogen bond, and Tyr aromatic ring interaction with copper ion is not as strong as for Phe aromatic ring.

It should also be emphasized, that the purpose of this work was to characterize the electrochemical properties of particular coordination forms of copper(II)-peptide complexes. Therefore, the experiments were carried out at different pH values, selected to ensure the predominance of a given form of each complex. However, more detailed studies (data not shown) indicated that the 4N form of the biologically relevant Cu(II)-NSFRY-NH_2_ complex dominated at physiological pH = 7.4 (~85% and ~15% of 4N and 3N species, respectively) and thus the Cu(II)/Cu(III) electrode process was mainly observed.

#### 3.2.3. Redox behavior of other complexes (with coordination less than 4)

The electrochemical characterization of the complexes less stable than 4N species was attempted, despite the fact that it is difficult to clearly identify 3N and 2N coordination structures on the basis of voltammetric measurements. Copper(II) ions complexed by AAAAA-NH_2_ ligand (pH = 8.0 indicates 3N coordination structure based on species distribution diagram, see Table 1), are oxidized and reduced at less positive potentials in comparison with other studied species (Fig 4). This redox process is less reversible and thermodynamically more favorable than for 3N form of Cu(II)-NSFRY-NH_2_, similarly to the behavior of 4N structures of Cu(II)-AAAAA-NH_2_ and Cu(II)-NSFRY-NH_2_. On the other hand, the reversibility of the redox process measured for Cu(II)-NSFRY-NH_2_ and Cu(II)-NSFRA-NH_2_ species is comparable. However, the peak current for the latter complex is significantly decreased and simultaneously a small pre-peak, corresponding to Cu(0)/Cu(II) process, appears at potential close to 0 mV (the determination of the accurate value of E_1/2_ was difficult). This supposition was justified by a typical adsorption peak observed during the experiments, which may result from the reduction of Cu(II) and copper deposition on the surface of the electrode [27,28]. More detailed analysis of the CV curves is difficult, since several complex forms remained at equilibrium at the given pH and the mixed effect of their presence was pronounced (according to the values of the protonation constants (pK_a_), calculated for various forms of Cu(II)-peptide complexes, it is possible to obtain an unique, predominant complex form in solution only in the case of 4N species) [13].

Slight lowering of pH of the solution led to the shift of complex equilibria towards 2N species, which causes the reduction of the electrochemical responses recorded for Cu(II)-peptide species at more positive potential values. Moreover, the peak current attributed to the Cu(0)/Cu(II) process is amplified (especially for both Cu(II)-NSFRY-NH_2_ and Cu(II)-NSFRA-NH_2_). Such results indicate that 2N complexes are relatively weak, since the application of a negative potential induces immediately the reduction of copper(II) center to the metallic form, which is reflected in the formation of adsorption peaks (Fig 5). Finally, the peak currents associated with the adsorption of copper bound to NSFRY-NH_2_ and NSFRA-NH_2_ ligands are comparable, whereas the corresponding peak current registered for Cu(II)-AAAAA-NH_2_ is significantly lower (Fig 5).

## 4. Conclusions

Electrochemical and structural studies of C-terminal tail of ANF peptide were presented in this work. Molecular modeling performed for 4N structure of Cu(II)-NSFRY-NH_2_ complex indicated that the four nitrogen atoms are involved in the formation of square planar structure with copper(II) ion. The cation-π interactions occurring between phenylalanine and arginine as well as between tyrosine and Cu(II) ions provided specific conformation defined as peptide box [18], that may alter the properties of the metal-peptide species. Detailed voltammetry experiments confirmed the highest stability of Cu(II)-NSFRF-NH_2_ and Cu(II)-NSFRY-NH_2_ complexes mainly due to the Phe-Arg cation-π interactions i.e. the formation of the shield above the coordination plane, protecting Cu(II)-peptide complex from hydrolysis (whereas the influence of Cu(II)-Tyr cation-π interactions was less pronounced). Many structural possibilities stabilizing the copper/nickel complexes were reported, and our molecular modelling shows, as a new in this matter, an additional, and omitted in the literature, intramolecular cation-π interaction between Phe-3 and Arg-4, which can play a crucial role in the electrochemical activity. According to the previous studies, the role of Asn as a first anchoring site for metal ion coordination was also important for complex stabilization. On other hand, AAAAA-NH_2_ peptide was found as the weakest chelator of copper(II) ions and the mechanism of the electron transfer was more complicated in the case of Cu(II)-AAAAA-NH_2_ complex. The results reported in this work are consistent with the stability of Cu(II) complexes of NSFRY peptide analogues provided by other instrumental techniques [11,12].

Concluding, several studies regarding the binding of copper(II) by NSFRY-NH_2_ and its analogues have been reported. However, as far as we know, the electrochemical techniques have been not introduced to assess the influence of cation-π interactions on the stability and redox properties of the complexes of copper(II) ions with NSFRY-NH_2_ analogues. The obtained results are promising for the elucidation of the relationship between structural and redox properties of active copper centers not only in Cu(II)-NSFRY-NH_2_ complexes, but also in various copper dependent proteins.
